# Fufang Duzhong Jiangu granule (FFDZ) ameliorates osteoarthritis development through maintaining subchondral bone homeostasis

**DOI:** 10.3389/fcell.2025.1610007

**Published:** 2025-08-06

**Authors:** Houfu Ling, Jianbo Xu, Qinghe Zeng, Zhen Fang, Liangyan Cheng, Wenhua Yuan, Jiali Chen, Yuliang Huang, Songfeng Hu, Hongting Jin, Peijian Tong, Ke Lu, Pinger Wang

**Affiliations:** ^1^ Institute of Orthopaedics and Traumatology, The First Affiliated Hospital of Zhejiang Chinese Medical University (Zhejiang Provincial Hospital of Chinese Medicine), Hangzhou, China; ^2^ Department of Orthopaedic Surgery, The First Affiliated Hospital of Zheiiang Chinese Medical University (Zhejiang Provincial Hospital of Chinese Medicine), Hangzhou, China; ^3^ The First People’s Hospital of Xiaoshan District, Xiaoshan Affiliated Hospital of Wenzhou Medical University, Hangzhou, China; ^4^ Department of the Rehabilitation, The Third Affiliated Hospital of Zhejiang Chinese Medical University, Hangzhou, China; ^5^ Department of the Orthopedic Surgery, The Second Affiliated Hospital of Zhejiang Chinese Medical University, Hangzhou, China; ^6^ Department of the Joint Surgery, Shaoxing Hospital of Traditional Chinese Medicine, Shaoxing, China; ^7^ Shenzhen Hospital, Southern Medical University, Shenzhen, China

**Keywords:** subchondral bone homeostasis, mesenchymal stem cell, TGF-β signaling, osteoarthritis, Fufang Duzhong Jiangu granule

## Abstract

**Introduction:**

Osteoarthritis (OA) is a widespread joint disorder predominantly marked by cartilage degeneration and the hardening of subchondral bone, with a lack of disease-modifying drugs for OA treatment. Fufang Duzhong Jiangu granule (FFDZ), a Chinese medicine, has demonstrated efficacy and safety in the clinical management of OA patients. However, the precise mechanisms through which it operates are still not fully understood.

**Methods:**

In this study, we set out to explore the protective effects of FFDZ on destabilization of the medial meniscus (DMM) surgery-induced OA mice and elucidate its mechanism underlying the delay of OA progression both *in vivo* and *in vitro*. The pathological alterations of OA in DMM-induced mice were examined by gait analysis, μCT, histopathology and immunohistochemistry.

**Results:**

We observed that FFDZ administration effectively attenuated cartilage degradation and subchondral bone deterioration at 8 weeks after DMM operation. Gait analysis indicated that FFDZ could alleviate OA pain caused by surgery. Notably, FFDZ exhibited a potent inhibitory effect on osteoclast activity, as evidenced by tartrate-resistant acid phosphatase (Trap) staining, and repressed the osteoblastic expression of osterix and alkaline phosphatase (ALP) increasing after DMM operation in subchondral bone area. Subsequently, we confirmed that FFDZ reduced the number of CD44^+^ and CD73^+^ mesenchymal stem cells (MSCs) and inhibited the phosphorylation level of Smad2 (pSmad2) in subchondral bone. Similarly, FFDZ also suppressed the activation of TGF-β signaling in MSCs.

**Discussion:**

In summary, this study demonstrated that FFDZ decelerated OA development in knee joints of mice after DMM potentially by maintaining subchondral bone homeostasis, providing evidences for the further application of FFDZ as an OA treatment.

## 1 Introduction

Osteoarthritis (OA) is a prevalent joint disorder impacting over 250 million people globally, making it one of the leading causes of disability worldwide. (Hunter and Bierma-Zeinstra, 2019). Due to the increasing prevalence of obesity and the aggravation of population aging, the morbidity of OA has been gradually increasing in recent years (Bortoluzzi et al., 2018). It is estimated that approximately 8% national population and more than 80% of people aged over 75 years suffer from OA in China, resulting in a dramatically high medical and socioeconomic burden (Liu et al., 2018). Despite its prevalence, there remains a significant lack of disease-modifying drugs to halt the progression of OA. This underscores the urgent need to explore novel therapeutic approaches for OA treatment. (Tong et al., 2022).

It is well-recognized that OA is a whole-joint disease, characterized by progressive cartilage degeneration, subchondral bone sclerosis, osteophyte formation, and synovial inflammation. (Berenbaum et al., 2018; Chen et al., 2017). Though, cartilage degeneration is regarded as the predominant feature of OA, the initial pathological changes in OA development still remains in controversy (Hu W. et al., 2021). In the past decades, it has been established that subchondral bone is also a crucial component that contribute to OA initiation and development (Coughlin and Kennedy, 2016). And, in clinic, subchondral bone marrow lesion (BML) was commonly observed in most OA patients, particularly those at an early-middle stage (Bowes et al., 2016). Subchondral bone homeostasis is mainly regulated by osteoblasts and osteoclasts under physiological condition (Jiang et al., 2022). However, under osteoarthritic conditions, abnormal mechanical loading triggers osteoclastogenesis, primarily due to the increased secretion of receptor activator of nuclear factor-κB ligand (RANKL) from osteocytes (Xiong et al., 2011; Zhu et al., 2019). Subsequently, the increased osteoclast-mediated bone resorption results in the release of matrix-embedded factors, such as transforming growth factor-beta (TGF-β), which in turn stimulates the migration and osteogenic differentiation of mesenchymal stem cells (MSCs) in the subchondral bone (Cai et al., 2020; Hu Y. et al., 2021). As a result, both enhanced activity of osteoclasts and osteoblasts contribute to high bone turnover while impairing subchondral bone homeostasis, subsequently disrupting the distribution of mechanical stress on articular cartilage and chondrocyte metabolism (Zhen et al., 2021). Furthermore, studies have demonstrated that inhibiting TGF-β signaling in subchondral bone can attenuate cartilage degeneration and slow down OA progression (Xie et al., 2016; Zhen et al., 2013).

Recently, emerging evidence has underscored the link between the subchondral bone microenvironment and OA pain (Hu W. et al., 2021; Temp et al., 2020). A significant presence of sensory nerves has been observed in the subchondral bone area, suggesting its potential role in regulating OA pain. Specifically, it has been demonstrated that subchondral bone osteoclasts exert an influence on sensory innervation and contribute to OA pain (Zhu et al., 2019). Moreover, a recent study has revealed that aberrant mechanical stress can stimulate subchondral bone osteoblast to secret prostaglandin E2 (PGE2), which subsequently induces sensitization of dorsal root ganglia (DRG) neurons through sodium channel Na_V_1.8, thereby mediating OA pain in mice (Zhu et al., 2020). These findings indicated that subchondral bone homeostasis mediated by osteoblasts and osteoclasts plays a vital role in the pathogenesis of OA pain as well as progression. Hence, maintaining subchondral bone homeostasis perhaps is a potential approach for OA treatment.

Fufang Duzhong Jiangu granule (FFDZ) is a Chinese medicine that has been used for the treatment of OA in China. Clinically, FFDZ is commonly used in the treatment of early-stage osteoarthritis, with no observed adverse effects. Although some studies have investigated the specific mechanisms of FFDZ in OA therapy, they remain incompletely elucidated (Wang et al., 2025). In this study, we aimed to validate the therapeutic effects of FFDZ and investigate its potential mechanisms both *in vivo* and *in vitro*. Our findings may offer further evidence supporting the extensive clinical application of FFDZ as a treatment for OA.

## 2 Materials and methods

### 2.1 Preparation and UPLC analysis of FFDZ

In this study, all FFDZ were purchased from the China Resources Double-Crane Pharmaceutical Co.,Ltd. (No.2011905). The main components of FFDZ were shown in Supplementary Table S1. Ultra-Performance Liquid Chromatography (UPLC) was employed to ensure the quality control of FFDZ and to accurately identify its chemical components. First, FFDZ was dissolved in pure water at a concentration of 1 g/mL and then further diluted to 400 mg/mL with methanol. After centrifugation at 12,000 rpm for 15 min, the supernatant was collected and filtered through a 0.22 μm microporous membrane to prepare the test solution. Then, a Titank C18 column (250 mm × 4.6 mm, 5 μm, Waters 2489, United States) was used to isolate the constituents of FFDZ at a flow rate of 1 mL/min. The mobile phase was composed of solvent A (water containing 0.5% acetonitrile) and solvent B (0.1% formic acid). The elution process lasted for 81 min, with the following gradient: 0–60 min, 5%–30% A, 60–70 min, 30%–95% A, 70–71 min, 95%–5% A. The column temperature was maintained at 25°C, and UV detection was performed at a wavelength of 274 nm. Finally, five components including calycosin-7-glucoside, gallic acid, albiflorin, pinecrosinol diglucoside, and geniposide were detected (Supplementary Figure S1).

### 2.2 Mice and OA modeling

The 36 male C57BL/6 mice (25 ± 5 g), aged 10 weeks, used in this experiment were purchased from the Experimental Animal Center of Zhejiang Chinese Medical University (Hangzhou, China). The mice were housed in cages under pathogen-free conditions, maintained on a 12-h light/dark cycle, and provided with free access to food and water. This study adhered to Chinese legislation on the use and care of laboratory animals and was approved by the Committee on the Ethics of Animal Experiments of Zhejiang Chinese Medical University (20221010-09). To establish the OA model, we performed destabilization of the medial meniscus (DMM) surgery (Glasson et al., 2007). Briefly, the medial meniscus instability was induced by transverse incision of the tibial meniscus ligament and the anterior meniscus angle in the right hind limb of anesthetized mice. All operations were under aseptic condition and antibiotics were used to prevent infection. The sham underwent a similar surgical operation with no damage to meniscotibial ligament.

### 2.3 Experimental animals grouping and drug administration

The 36 mice were randomly allocated into two treatment cohorts: 18 mice for the 4-week treatment group and 18 mice for the 8-week treatment group. After establishing the surgical model of OA, the mice were divided into three groups randomly, with six mice per group. Both the DMM group and the FFDZ group underwent DMM surgery, whereas the sham group underwent a similar procedure without transecting the meniscotibial ligament. Mice were harvested after four or 8 weeks of treatment respectively.

Drug administration began on the day after DMM surgery. A solution of 36 g FFDZ dissolved in 50 mL of normal saline was orally administered to the mice in the FFDZ group at a dosage of approximately 0.15 mL/10 g body weight, following the human-to-mouse equivalent dosage conversion, for either four or eight consecutive weeks. The sham and DMM groups received an equal volume of normal saline for the same duration.

### 2.4 Gait analysis

After 8 weeks treatment, DigiGait imaging system (Mouse Specifics) was utilized to record and analyze the gait changes of each group. In brief, a transparent flat treadmill was operated at a specific speed of 18 cm/s. A video camera positioned beneath the treadmill captured ventral images of the mice as they ran. These images were then processed by a computer to generate paw data. Each measurement session lasted up to 30 s, with 5-s segments (containing more than 10 consecutive strides) used for analysis. The following parameters of the surgical hind limb were observed: paw area, stride length, swing time, and stance time.

### 2.5 Micro-CT analysis

All mice were euthanized by CO_2_, and their right knee joints were harvested after four or 8 weeks of treatment. Micro-computed tomography (Micro-CT) (Skyscan 1176, Bruker, Kontich, Belgium) was then employed to analyze the bone microstructural changes in the knee joints. The region of interest was defined as the area between the proximal tibia growth plate and the tibial plateau. The following parameters were collected for analysis: Percent bone volume (BV/TV, %), Trabecular thickness (Tb.Th, mm), Trabecular number (Tb.N, 1/mm), and Trabecular separation (Tb.Sp, mm).

### 2.6 Histological analysis

Samples were first fixed in 4% paraformaldehyde for 3 days and then decalcified with 14% EDTA solution for 20 days. Following this, the samples were embedded in paraffin and sectioned at 3 μm thickness at the medial compartment of the joints for ABH (Alcian Blue Hematoxylin/Orange G) and SO (Safranin O/Fast Green) staining. Histomorphometric analysis was conducted using OsteoMeasure software (Decatur, GA). The grade of OA progression was assessed through double-blind observation in accordance with the recommendations of the Osteoarthritis Research Society International (OARSI).

### 2.7 Immunohistochemistry staining

Immunohistochemistry was performed to observe the expression of specific proteins. Briefly, the deparaffinized sections were immersed in 0.3% hydrogen peroxide to block endogenous peroxidase activity, followed by blocking with normal goat serum (diluted 1:20) for 20 min at room temperature. Subsequently, primary antibodies were added and incubated overnight at 4°C. The next day, the sections were treated with secondary antibodies for 30 min, and positive staining was visualized using diaminobenzidine solution (Invitrogen, MD, United States). Counterstaining was performed with hematoxylin for 5 s. The following antibodies were used in this study: anti-Col2 (ab34712, 1:200), anti-Aggrecan (NB100-74350, 1:200), anti-MMP-13 (ab39012, 1:200), anti-Adamts5 (bs-3573R, 1:200), anti-ALP (ARG57433, 1:150), anti-Osterix (ER1914-47, 1:300), anti-CD44 (ab243894, 1:500), anti-CD73 (RLT5254, 1:500), anti-pSmad2 (ab188334, 1:250).

### 2.8 Preparation of FFDZ drug serum

Thirty 12-week-old male SD rats (200 ± 30 g), obtained from the Experimental Animal Center of Zhejiang Chinese Medical University, were randomly assigned to two groups (n = 15 per group). The rats in the FFDZ group received FFDZ treatment (3.8 g/kg body weight) for seven consecutive days, while the control group received an equivalent dosage of normal saline for the same duration. Following the last treatment over 2 hours later, all rats were euthanized for blood sampling. Serum was collected by centrifugation at 3,000 rpm, then filtered and inactivated at 56°C for 30 min before being stored at −80°C.

### 2.9 Cell treatment and Western blot

The mesenchymal stem cell line C3H10T1/2 (ATCC, Manassas, VA, United States) was utilized for *in vitro* experiments. The cells were cultured in Dulbecco’s Modified Eagle Medium (Gibco, MD, United States) supplemented with 10% fetal bovine serum (FBS) (Sigma, MO, United States) and 1% penicillin-streptomycin (Gibco, MD, United States) at 37°C in a 5% CO_2_ atmosphere. Cultured cells were treated with TGF-β receptor inhibitor (SB505124) or FFDZ containing drug serum (concentration of 10%, 15%, 20%) for 24 h, 48 h and then exposed to TGF-β1 (10 ng/μL) for 30 min after 4 h starvation and processed for Western Blot. The appropriate RIPA lysis buffer was added to the C3H10T1/2 cells, which were then gently and repeatedly agitated on ice for 30 min. The mixture was subsequently centrifuged at 12,000 rpm for 10 min at 4°C to obtain the supernatant. The protein concentration of each group was measured using a BCA protein quantification kit. A specific volume of 5 × loading buffer was then added to the remaining protein supernatant, followed by denaturation at 100°C for 5 min.

Proteins (20 μg per lane) were separated using an 8% SDS-PAGE gel and subsequently transferred onto a PVDF membrane. After blocking for 1 hour with 5% skim milk, the membranes were incubated overnight at 4°C with primary antibodies: Smad2 (1:1000 dilution, Abcam), p-Smad2 (1:1000 dilution, Abcam), and β-actin (1:3000 dilution, Sigma-Aldrich). The next day, the membranes were incubated with the appropriate secondary antibodies for 1 hour at room temperature. Protein bands were visualized using the ImageQuant LAS 4000 system (EG, United States). The acquired bands were analyzed with Image Lab™ software (Bio-Rad) and normalized to β-actin.

### 2.10 Statistical analysis

All data are presented as mean ± standard deviation. Group means were compared using one-way analysis of variance (ANOVA) followed by Tukey’s *post hoc* test. A P value of less than 0.05 was considered statistically significant. Statistical analyses were conducted using GraphPad Prism software version 8.0.

## 3 Results

### 3.1 FFDZ attenuated cartilage degeneration in DMM-induced OA mice

**FIGURE 1:**
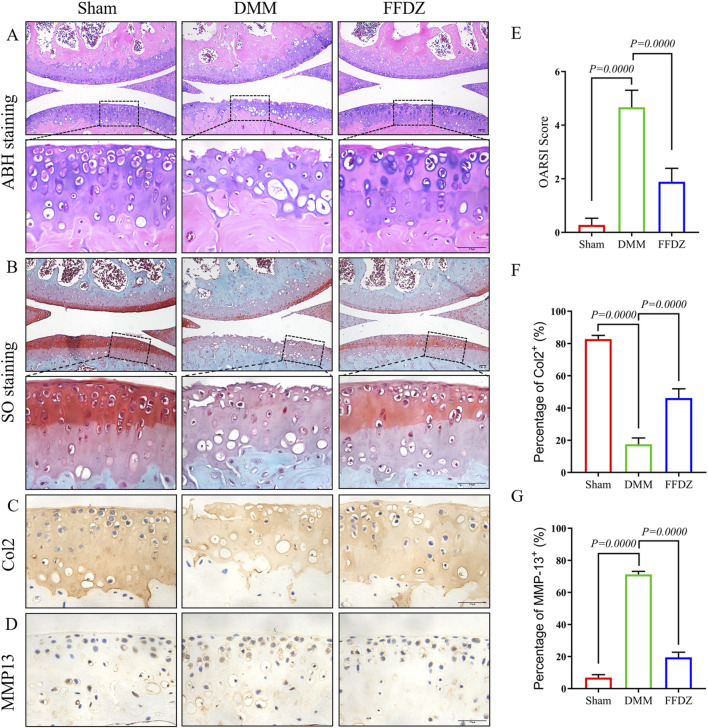
FFDZ effectively reduced cartilage degeneration in mice with DMM-induced osteoarthritis at 8 weeks. **(A)** ABH staining and **(B)** Safranin O staining of surgical knee in WT mice. Immunohistochemical staining of Col2 **(C)** and MMP13 **(D)** expression in cartilage at 8 weeks. Scale bar = 100 μm. **(E)** OARSI scoring of the sections analyzed by histomorphometry. **(F,G)** Percentage of Col2 and MMP13 positive expression. Data are presented as means ± SD (n = 6). The specific p-values are clearly labeled in the figure.

To investigate whether FFDZ has a positive effect on disease activity and development in OA, we administered FFDZ orally in mice after DMM. Specifically, ABH staining and safranin O staining were used to assess the effect of FFDZ on articular cartilage after 8 weeks treatment. As shown in Figures 1A,B, the DMM-induced OA mice shown obvious cartilage degeneration and abrasion compared with the sham group. However, the mice treated with FFDZ exhibited retention of cartilage integrity and proteoglycan (Figures 1A,B). The OARSI score of the DMM-induced OA mice was significantly higher than sham mice, but decreased after treatment with FFDZ (Figure 1E). Furthermore, compared with the sham mice, the expression of Col2 in DMM-induced OA mice was downregulated but MMP13 was upregulated significantly, and the Col2 was upregulated and MMP13 was downregulated in FFDZ mice as expected (Figures 1C,D). Quantitation of Col2 and MMP13 positive cells in cartilage were consistent with the immunohistochemistry staining (Figures 1F,G). These results confirmed the therapeutic effect of FFDZ on cartilage degradation in mice with OA.

### 3.2 FFDZ alleviated subchondral bone sclerosis and OA pain in DMM-induced mice

**FIGURE 2:**
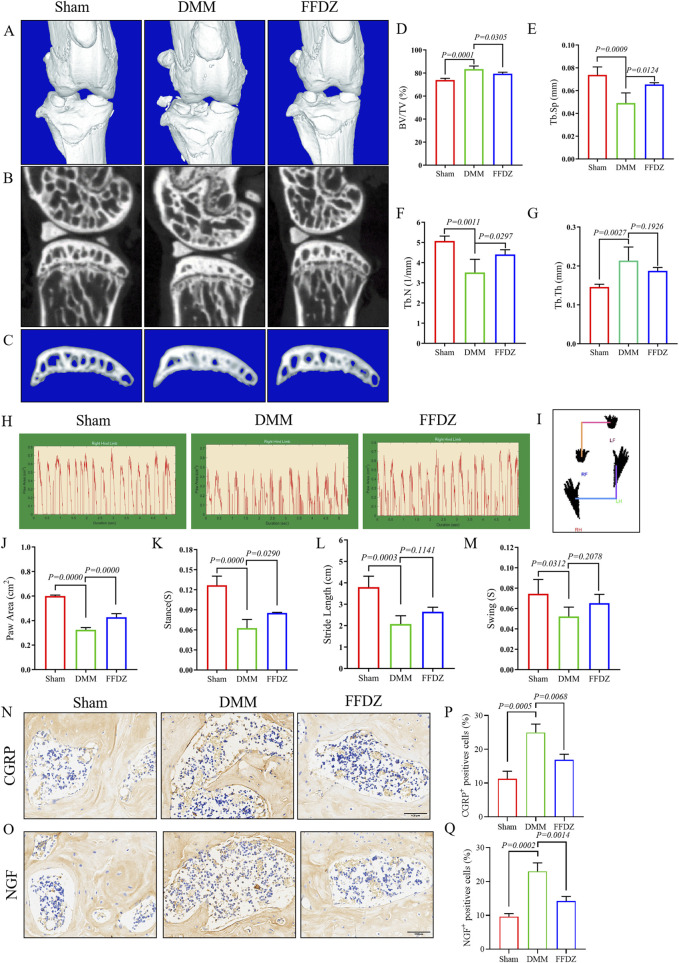
FFDZ alleviated subchondral bone sclerosis and OA-related pain in DMM mice at 8 weeks μCT analysis was conducted to examine the changes in bone structure. **(A–C)** Representative 3D reconstructions of the surgical knee joint and subchondral bone. Quantitative analysis of the subchondral bone structure **(D)** BV/TV (%), **(E)** Tb.Sp (mm), **(F)** Tb.N (1/mm) and **(G)** Tb.Th (mm). Gait analysis was conducted using the DigiGait imaging system, **(H)** the dynamic giat signals of paw area in each group, **(I)** the posture plot of mice. **(J)** Paw area (cm^2^), **(K)** Stance (s), **(L)** Stride length (cm) and **(M)** Swing (s) of the right hind limb were chosen as the observation index. Immunohistochemical staining of CGRP **(N)** and NGF **(O)** expression in cartilage at 8 weeks. Scale bar = 100 μm. **(P,Q)** Percentage of CGRP and NGF positive expression. The Data are presented as means ± SD (n = 6). The specific p-values are clearly labeled in the figure.

Then, we utilized Micro-CT imaging to investigate the potential impact of FFDZ on the pathological progression of subchondral bone in OA condition. In the 3D reconstruction of the knee joints, obvious subchondral bone osteosclerosis was observed in mice with OA (Figures 2A–C). Based on Micro-CT analysis data, in DMM-induced mice, the bone microarchitectures including BV/TV, Tb.Th were significant increased, and the Tb.N, Tb.Sp were significant decreased compared with the sham mice (Figures 2D–G). However, the phenotype of subchondral bone sclerosis in response to DMM operation was obviously alleviated by FFDZ administration (Figures 2A–C), as supported by the decreased BV/TV, Tb.Th and increased Tb.N, Tb.Sp compared to DMM-induced mice (Figures 2D–G). In addition, we also observed that FFDZ could partially ameliorated the gait disturbance induced by DMM, as evidenced by an increase in paw area and stance time (Figures 2H–M), which indicated that FFDZ treatment reduced OA pain in DMM-induced mice. Moreover, in the immunohistochemical analysis of the two pain markers CGRP and NGF, we demonstrated that FFZD effectively attenuates the aberrant expression induced by the DMM model (Figures 2N–Q). In brief, the above results demonstrated FFDZ could effectively attenuate the progression of OA in mice after treatment.

### 3.3 FFDZ preserved cartilage integrity in DMM-induced mice

**FIGURE 3:**
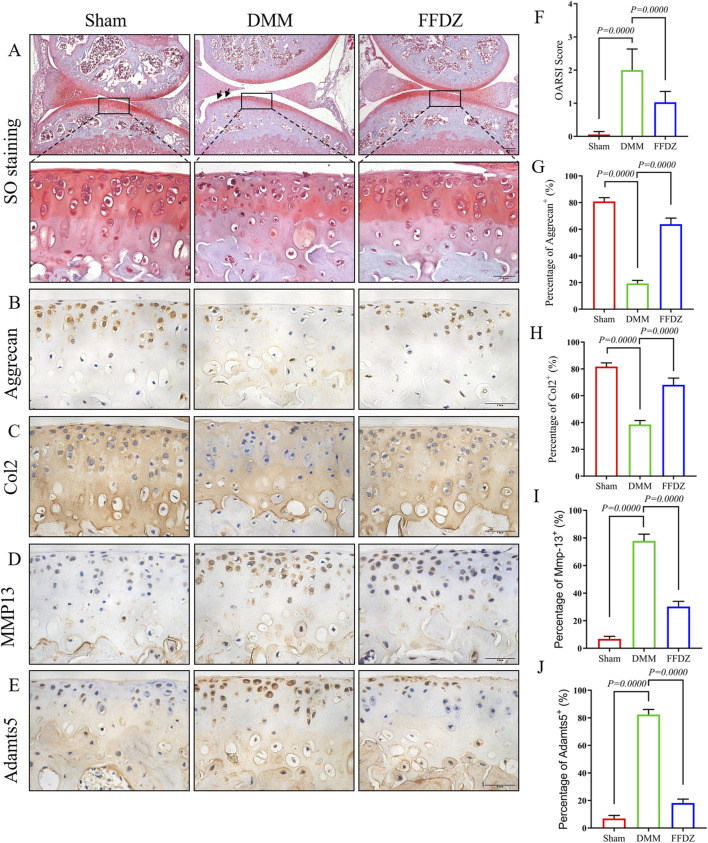
FFDZ preserved cartilage integrity in DMM mice at 4 weeks. **(A)** Safranin O staining of surgical knee in mice at 4 weeks. **(B–E)** Immunohistochemical staining of Aggrecan, Col2, MMP13 and Adamts5 in cartilage at 4 weeks, Scale bar = 100 μm. **(F)** OARSI scoring of each group. **(G–J)** Percentage of positive expression of Aggrecan, Col2, MMP13 and Adamts5. Data are presented as means ± SD (n = 6). The specific p-values are clearly labeled in the figure.

The positive effect of FFDZ on OA progression was observed after 8 weeks treatment, both in articular cartilage and subchondral bone. Then we sought to investigate these protective effects of FFDZ on DMM-induced OA mice at earlier stage. Thus, mice were sacrificed for histological analysis and IHC staining after a 4-week treatment with FFDZ. As the safranin O staining showed (Figure 3A), much more proteoglycan loss was observed in DMM-induced mice compared to mice that received a sham operation. As expected, the administration of FFDZ effectively preserved the structural integrity of articular cartilage induced by DMM surgery, and OARSI score was consistent with the staining result (Figure 3F). Correspondingly, we also examined the role of FFDZ in anabolic and catabolic metabolism in cartilage. Specifically, the expression of Col2 and Aggrecan were downregulated while MMP13 and Adamts5 were upregulated in DMM-induced mice (Figures 3B–E). Of note, compared with DMM-induced mice, the expression of Col2 and Aggrecan in FFDZ mice were significantly upregulated (Figures 3B,C). And the expression of MMP13 and Adamts5 were much lower than that in DMM-induced mice at 4 weeks after surgery (Figures 3D,E). The quantification of Col2, Aggrecan, MMP13 and Adamts5 positive cells further demonstrated the chondroprotective effect of FFDZ on OA (Figures 3G–J). Taken together, these data suggested that FFDZ could protect articular cartilage against DMM-induced OA.

### 3.4 FFDZ modulated subchondral bone homeostasis at early-middle stage of OA

**FIGURE 4:**
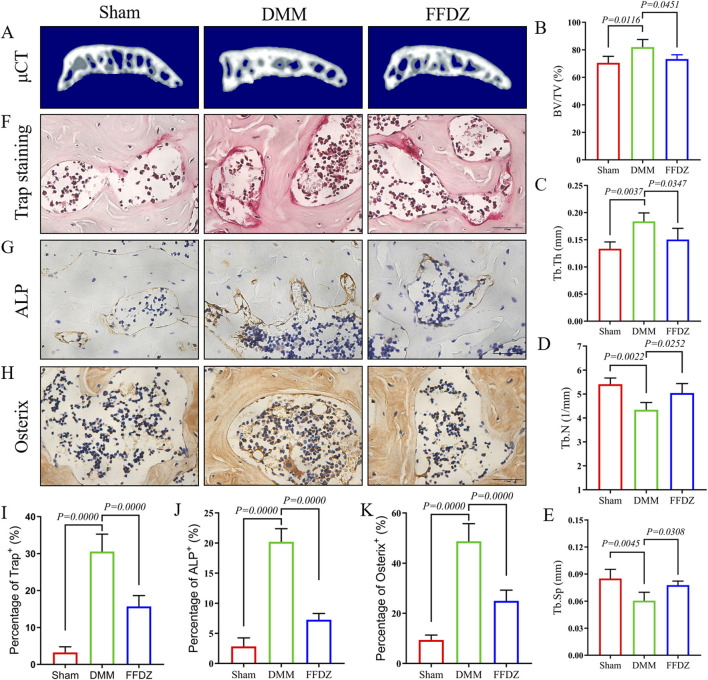
FFDZ modulated subchondral bone homeostasis at early-middle stage of OA. **(A)** 3D reconstruction of the subchondral bone of each group at 4 weeks. **(B–E)** Quantitative analysis of the subchondral bone BV/TV (%), Tb.Th (mm), Tb.N (1/mm) and Tb.Sp (mm). **(F–H)** Immunohistochemical staining of Trap, ALP and Osterix in subchondral bone at 4 weeks, Scale bar = 100 μm. **(I–K)** Quantification of the positive expression of Trap, Alp and Osterix (%). Data are presented as means ± SD (n = 6). The specific p-values are clearly labeled in the figure.

Then, mild subchondral bone osteosclerosis was detected through Micro-CT analysis in DMM-induced mice (Figure 4A), with the increase of BV/TV, Tb.Th and decrease of Tb.N, Tb.Sp (Figures 4B–E). However, the 3D construction of subchondral bone in FFDZ showed slighter osteoclerosis than DMM-induced mice, which was closer to the sham mice. Compared with the DMM-induced mice, BV/TV, Tb.Th were significantly decreased and Tb.N, Tb.Sp were increased in mice treated with FFDZ (Figures 4A–E). According the Micro-CT analysis, we further investigated the osteoclastic and osteoblastic activity in subchondral bone to determine the subchondral bone metabolism. Interestingly, both osteoclastic and osteoblastic activity were enhanced in subchondral bone at 4 weeks post DMM surgery, as revealed by the increased number of tartrate-resistant acid phosphatase (Trap)^+^ osteoclast cells, osterix^+^ and ALP^+^ cells (Figures 4F–H). Furthermore, FFDZ effectively modulated the activity of osteoclast and osteoblast cells in subchondral bone, showed in the significant downregulation of Trap^+^ osteoclast cells, osterix^+^ and ALP^+^ cells (Figures 4F–K). These findings illustrated that FFDZ had the potential to sustain coordinated remodeling of the subchondral bone.

### 3.5 FFDZ inhibited the excessive activation of TGF-β signaling in MSCs

**FIGURE 5:**
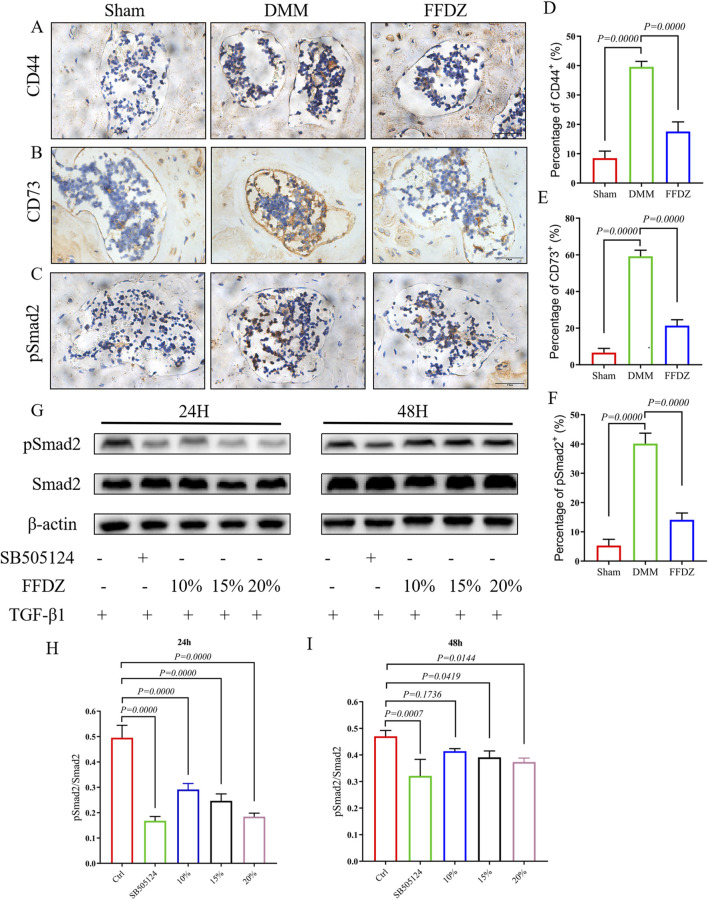
FFDZ inhibited the excessive activation of TGF-β signaling in MSCs. **(A–C)** Immunohistochemical staining of CD44, CD73 and pSmad2 in subchondral bone at 4 weeks, Scale bar = 100 μm. **(D–F)** Quantification of the positive expression of CD44, CD73 and pSmad2 (%). **(G–I)** Effects of FFDZ on the activation of the TGF-β signaling pathway in C3H10T1/2 cells. **(A–F)** Data are presented as means ± SD (n = 6), **(G–I)** Data are presented as means ± SD (n = 6). The specific p-values are clearly labeled in the figure.

According to prior studies (Qiu et al., 2010; Zhen et al., 2013), mesenchymal stem cells (MSCs) located in the subchondral bone are essential in regulating the remodeling of subchondral bone. In this study, we examined the expression of CD44^+^ and CD73^+^ MSCs in the subchondral bone at 4 weeks after DMM surgery. The IHC staining showed that FFDZ significantly attenuated the increase in the number of CD44^+^ and CD73^+^ MSCs in the subchondral bone compared to DMM (Figures 5A,B,D,E). Since high levels of active TGF-β contribute to recruiting MSCs into the subchondral bone marrow, leading to aberrant bone formation and subchondral bone sclerosis (Yu et al., 2020; Zhao et al., 2016). Then we investigated whether FFDZ had inhibitory effects on TGF-β signaling in MSCs. Importantly, we found that FFDZ treatment clearly inhibited the increased expression of phospho-Smad2 (pSmad2) observed in response to DMM surgery within the subchondral bone (Figures 5C,F). Furthermore, Western Blot analysis of MSCs also confirmed that FFDZ dose-dependently blocked Smad2 phosphorylation after 24 h of treatment; however, only doses of 15% or 20% downregulated pSmad2 expression after 48 h of intervention (Figures 5G–I). This suggests that FFDZ acts as an inhibitor of canonical TGF-β signaling for MSCs. Thus, these results from both *in vivo* and *in vitro* studies indicate that FFDZ maintains subchondral bone homeostasis, at least in part, through regulating TGF-β signaling in MSCs, resulting in reduced abnormal subchondral bone formation.

## 4 Discussion

Osteoarthritis (OA) is a chronic, progressive degenerative disorder marked by the deterioration of cartilage, subchondral bone sclerosis, and inflammation of the synovial tissue (Soul et al., 2021). Currently, there are no widely recognized effective disease-modifying drugs for treating OA due largely to limited comprehension of its pathogenesis (Chen et al., 2017; Tong et al., 2022). Notably, OA remains a persistent disturbance that substantially diminishes patients’ quality of life. (Hunter et al., 2020). Therefore, it is urgent to identify new therapeutic targets and explore potential treatments that can efficiently and safely attenuate the development of OA (Zhang et al., 2016).

FFDZ is a traditional Chinese medicine formula known for its effective alleviation of OA symptoms in clinical settings. This study aims to validate the therapeutic effects of FFDZ on mice with DMM-induced OA at various stages of the disease. Consistent with clinical findings, we found that FFDZ could decelerate cartilage degeneration and subchondral bone sclerosis both at 4 or 8 weeks post-operation in mice. Experimental results further demonstrated that FFDZ modulated the subchondral bone homeostasis by inhibiting the excessive bone remodeling mediated by osteoblasts and osteoclasts. Furthermore, we observed that FFDZ attenuated the increased number of CD44^+^ and CD73^+^ MSCs in the subchondral bone, and downregulated the expression of pSmad2, the pivot regulator of canonical TGF-β signaling pathway. In summary, our data provide evidence that FFDZ attenuates OA progression possibly through inhibition of excessive TGF-β activity in recruiting MSCs for abnormal bone formation.

Despite substantial effort devoted to exploring the mechanism of OA, in the past decade, the occurrence of cartilage destruction and subchondral sclerosis during OA development is still a controversial issue (Hu Y. et al., 2021; Jiang et al., 2022). Accumulating evidence has provided insights into the role of subchondral bone microenvironment as a crucial regulator in OA pathogenesis (Cui et al., 2022; Hislop et al., 2022). In the early-middle stage of OA, joint instability disrupts the mechanical distribution and caused subchondral bone micro-damage even micro-fracture, which then caused excessive bone remodeling mediated by osteoclast and osteoblast (Taheri et al., 2023). In the present study, consistent with previous researches (Su et al., 2020; Sun et al., 2021), we found that DMM induction gave rise to a high bone turnover as revealed by the increased expression of Trap, osterix, and ALP in subchondral bone. Importantly, in this study, we observed concurrent activation of both osteoclasts and osteoblasts at 4 weeks post-DMM surgery. FFDZ was found to regulate osteoclastic and osteoblastic activity, ultimately restoring the subchondral bone microarchitecture to a level comparable to that in sham-operated mice. These findings suggest that FFDZ attenuates OA progression, at least partially, through subchondral bone remodeling in mice. As previously reported (Dong et al., 2016; Qin et al., 2019; Saito et al., 2010), inhibition of stromal cell-derived factor 1 (SDF-1)/C-X-C chemokine receptor type 4 (CXCR4) signaling or hypoxia-inducible factor-2α (HIF-2α) can relieve cartilage damage and OA progression. Thus, targeting subchondral bone might be employed as an alternative approach for cartilage degeneration as well as OA treatment.

In addition, given that bone formation is mainly regulated by osteoblast which is differentiated from MSCs, in this study, we further determined whether FFDZ could reduce the number of MSCs in subchondral bone. As expected, we found CD44^+^ and CD73^+^ MSCs in subchondral bone from mice treated with FFDZ was much less than that in DMM mice. Excessive activation of TGF-β signaling would induce uncoupled bone remodeling, which may result in the occurrence and development of OA (Cui et al., 2016; Zhen et al., 2013). Regardless of bone mass, TGF-β present in the bone matrix is released and activated during osteoclast-mediated bone resorption as part of coupled subchondral bone remodeling (Xian et al., 2012; Xiong et al., 2011). The Smad2/3-dependent TGF-β signaling pathway induces mesenchymal stem cells (MSCs) in the perivascular niche to migrate to the bone surface, where they differentiate into osteoblasts (Kusumbe et al., 2014). In a pathological state, excessive activation of osteoclast induced the excessive release and activation of TGF-β would disrupt the coupled bone remodeling, mediating the recruitment of MSCs to increase bone formation in bone marrow (Cai et al., 2020; Cui et al., 2022; Muratovic et al., 2019). In the present study, increased bone mass in the subchondral bone was observed in DMM mice, as reflected by elevated BV/TV and Tb.Th, along with reduced Tb.N and Tb.Sp. In addition, the number of CD44^+^ and CD73^+^ MSCs in the subchondral bone were increased in DMM mice, accompanied with upregulated expression of pSmad2. Notably, we found FFDZ could decrease the recruitment of CD44^+^ and CD73^+^ MSCs in subchondral bone which probably attributed to inhibition of TGF-β signaling in MSCs.

Also, there are several works have reported that OA pain is associated with subchondral bone microenvironment and it has been suggested that both osteoclast and osteoblast could secrete factors regulating sensory nerve sensitization or activation (Sun et al., 2022; Zhen et al., 2022). Specifically, a recent study has showed that, in OA condition, osteoclasts increased secretion of netrin-1 to induce sensory nerve axonal growth in subchondral bone and both genetic and pharmacologic inhibition of osteoclast activity could reduce the sensory innervation and pain behavior in OA mice (Zhu et al., 2019). Meanwhile, another research illustrated that osteoblast is able to produce neuromodulator, PGE2, which irritated sensory nerve fiber and further induced OA pain (Zhu et al., 2020). Additionally, intermittent parathyroid hormone (iPTH) was able to improve subchondral bone microstructure, decrease the expression of PGE2, and ameliorate OA pain (Sun et al., 2021). These results strongly indicated that aberrant subchondral bone homeostasis is responsible for OA pain and these findings provide a novel insight into the source of OA pain. Here, our gait analysis exhibited that administration with FFDZ effectively reduce pain induced by DMM operation. This result may be partially explained by the effect of FFDZ on maintaining subchondral bone homeostasis, specifically osteoblastic activity.

Since FFDZ was a formula with specific composition largely unknown, in this study, we also performed UPLC to control the quality and identify the accurate component of FFDZ. Herein, we observed five components containing calycosin-7-glucoside, gallic acid, albiflorin, pinecrosinol diglucoside, and geniposide. Similar to our results, a previous study has reported that geniposide had a protective effect on OA (Chen et al., 2018). Moreover, gallic acid isolated from *Cornus officinalis* could inhibit RANKL-induced osteoclastogenesis and regulate bone homeostasis via blocking Akt/ERK/JNK pathways (Zhang et al., 2022). And recent research with combination of pharmacology and animal experiments demonstrated pinecrosinol diglucoside had an OA-protective effect associated with PI3K/AKT signaling pathway in rabbit (Lou et al., 2022). These findings indicated that Chinese medicine may be a potential resource pool for OA drug research. However, the effect of these components on the subchondral bone homeostasis under OA condition is needed further investigation.

In conclusion, this study is the first to demonstrate that FFDZ can mitigate DMM-induced OA features, including articular cartilage degeneration, subchondral bone sclerosis, and pain. This effect may be achieved through the inhibition of excessive TGF-β activity, which otherwise recruits MSCs for aberrant bone formation. These findings provide a basis for the potential clinical application of FFDZ as a treatment for OA.

## Data Availability

The data that support the findings of this study are available from the corresponding author upon reasonable request.

## References

[B1] Berenbaum F. Wallace I. J. Lieberman D. E. Felson D. T. (2018). Modern-day environmental factors in the pathogenesis of osteoarthritis. Nat. Rev. Rheumatol. 14 (11), 674–681. 10.1038/s41584-018-0073-x 30209413

[B2] Bortoluzzi A. Furini F. Scirè C. A. (2018). Osteoarthritis and its management - epidemiology, nutritional aspects and environmental factors. Autoimmun. Rev. 17 (11), 1097–1104. 10.1016/j.autrev.2018.06.002 30213694

[B3] Bowes M. A. McLure S. W. Wolstenholme C. B. Vincent G. R. Williams S. Grainger A. (2016). Osteoarthritic bone marrow lesions almost exclusively colocate with denuded cartilage: a 3D study using data from the osteoarthritis initiative. Ann. Rheum. Dis. 75 (10), 1852–1857. 10.1136/annrheumdis-2015-208407 26672065

[B4] Cai G. Otahal P. Cicuttini F. Wu F. Munugoda I. P. Jones G. (2020). The association of subchondral and systemic bone mineral density with osteoarthritis-related joint replacements in older adults. Osteoarthr. Cartil. 28 (4), 438–445. 10.1016/j.joca.2020.02.832 32119971

[B5] Chen D. Shen J. Zhao W. Wang T. Han L. Hamilton J. L. (2017). Osteoarthritis: toward a comprehensive understanding of pathological mechanism. Bone Res. 5, 16044. 10.1038/boneres.2016.44 28149655 PMC5240031

[B6] Chen Y. Shou K. Gong C. Yang H. Yang Y. Bao T. (2018). Anti-inflammatory effect of geniposide on osteoarthritis by suppressing the activation of p38 MAPK signaling pathway. Biomed. Res. Int. 2018, 8384576. 10.1155/2018/8384576 29682561 PMC5846349

[B7] Coughlin T. R. Kennedy O. D. (2016). The role of subchondral bone damage in post-traumatic osteoarthritis. Ann. N. Y. Acad. Sci. 1383 (1), 58–66. 10.1111/nyas.13261 27671712

[B8] Cui Z. Crane J. Xie H. Jin X. Zhen G. Li C. (2016). Halofuginone attenuates osteoarthritis by inhibition of TGF-β activity and H-type vessel formation in subchondral bone. Ann. Rheum. Dis. 75 (9), 1714–1721. 10.1136/annrheumdis-2015-207923 26470720 PMC5013081

[B9] Cui Z. Wu H. Xiao Y. Xu T. Jia J. Lin H. (2022). Endothelial PDGF-BB/PDGFR-β signaling promotes osteoarthritis by enhancing angiogenesis-dependent abnormal subchondral bone formation. Bone Res. 10 (1), 58. 10.1038/s41413-022-00229-6 36031625 PMC9420732

[B10] Dong Y. Liu H. Zhang X. Xu F. Qin L. Cheng P. (2016). Inhibition of SDF-1α/CXCR4 signalling in subchondral bone attenuates post-traumatic osteoarthritis. Int. J. Mol. Sci. 17 (6), 943. 10.3390/ijms17060943 27322244 PMC4926476

[B11] Glasson S. S. Blanchet T. J. Morris E. A. (2007). The surgical destabilizationof the medial meniscus (DMM) model of osteoarthritis in the 129/SvEv mouse. Osteoarthr. Cartil. 15 (9), 1061–1069. 10.1016/j.joca.2007.03.006 17470400

[B12] Hislop B. D. Devine C. June R. K. Heveran C. M. (2022). Subchondral bone structure and synovial fluid metabolism are altered in injured and contralateral limbs 7 days after non-invasive joint injury in skeletally-mature C57BL/6 mice. Osteoarthr. Cartil. 30 (12), 1593–1605. 10.1016/j.joca.2022.09.002 PMC9671828 36184957

[B13] Hu W. Chen Y. Dou C. Dong S. (2021a). Microenvironment in subchondral bone: predominant regulator for the treatment of osteoarthritis. Ann. Rheum. Dis. 80 (4), 413–422. 10.1136/annrheumdis-2020-218089 33158879 PMC7958096

[B14] Hu Y. Chen X. Wang S. Jing Y. Su J. (2021b). Subchondral bone microenvironment in osteoarthritis and pain. Bone Res. 9 (1), 20. 10.1038/s41413-021-00147-z 33731688 PMC7969608

[B15] Hunter D. J. Bierma-Zeinstra S. (2019). Osteoarthr. Lancet 393 (10182), 1745–1759. 10.1016/S0140-6736(19)30417-9 31034380

[B16] Hunter D. J. March L. Chew M. (2020). Osteoarthritis in 2020 and beyond: a lancet commission. Lancet 396 (10264), 1711–1712. 10.1016/S0140-6736(20)32230-3 33159851

[B17] Jiang W. Jin Y. Zhang S. Ding Y. Huo K. Yang J. (2022). PGE2 activates EP4 in subchondral bone osteoclasts to regulate osteoarthritis. Bone Res. 10 (1), 27. 10.1038/s41413-022-00201-4 35260562 PMC8904489

[B18] Kusumbe A. P. Ramasamy S. K. Adams R. H. (2014). Coupling of angiogenesis and osteogenesis by a specific vessel subtype in bone. Nature 507 (7492), 323–328. 10.1038/nature13145 24646994 PMC4943525

[B19] Liu Q. Wang S. Lin J. Zhang Y. (2018). The burden for knee osteoarthritis among Chinese elderly: estimates from a nationally representative study. Osteoarthr. Cartil. 26 (12), 1636–1642. 10.1016/j.joca.2018.07.019 30130589

[B20] Lou H. Zhang Y. Fang J. Jin Y. (2022). Network pharmacology-based prediction and verification of the potential targets of pinoresinol diglucoside for OA treatment. Evid. Based Complement. Altern. Med. 2022, 9733742. 10.1155/2022/9733742 PMC9034917 35469160

[B21] Muratovic D. Findlay D. M. Cicuttini F. M. Wluka A. E. Lee Y. R. Edwards S. (2019). Bone marrow lesions in knee osteoarthritis: regional differences in tibial subchondral bone microstructure and their association with cartilage degeneration. Osteoarthr. Cartil. 27 (11), 1653–1662. 10.1016/j.joca.2019.07.004 31306782

[B22] Qin H. J. Xu T. Wu H. T. Yao Z. L. Hou Y. L. Xie Y. H. (2019). SDF-1/CXCR4 axis coordinates crosstalk between subchondral bone and articular cartilage in osteoarthritis pathogenesis. Bone 125, 140–150. 10.1016/j.bone.2019.05.010 31108241

[B23] Qiu T. Wu X. Zhang F. Clemens T. L. Wan M. Cao X. (2010). TGF-Beta type II receptor phosphorylates PTH receptor to integrate bone remodelling signalling. Nat. Cell. Biol. 12 (3), 224–234. 10.1038/ncb2022 20139972 PMC3704184

[B24] Saito T. Fukai A. Mabuchi A. Ikeda T. Yano F. Ohba S. (2010). Transcriptional regulation of endochondral ossification by HIF-2alpha during skeletal growth and osteoarthritis development. Nat. Med. 16 (6), 678–686. 10.1038/nm.2146 20495570

[B25] Soul J. Barter M. J. Little C. B. Young D. A. (2021). OATargets: a knowledge base of genes associated with osteoarthritis joint damage in animals. Ann. Rheum. Dis. 80 (3), 376–383. 10.1136/annrheumdis-2020-218344 33077471 PMC7892386

[B26] Su W. Liu G. Liu X. Zhou Y. Sun Q. Zhen G. (2020). Angiogenesis stimulated by elevated PDGF-BB in subchondral bone contributes to osteoarthritis development. JCI Insight 5 (8), e135446. 10.1172/jci.insight.135446 32208385 PMC7205438

[B27] Sun Q. Li G. Liu D. Xie W. Xiao W. Li Y. (2022). Peripheral nerves in the tibial subchondral bone: the role of pain and homeostasis in osteoarthritis. Bone Jt. Res. 11 (7), 439–452. 10.1302/2046-3758.117.BJR-2021-0355.R1 PMC9350689 35775136

[B28] Sun Q. Zhen G. Li T. P. Guo Q. Li Y. Su W. (2021). Parathyroid hormone attenuates osteoarthritis pain by remodeling subchondral bone in mice. Elife 10, e66532. 10.7554/eLife.66532 33646122 PMC8012060

[B29] Taheri S. Yoshida T. Böker K. O. Foerster R. H. Jochim L. Flux A. L. (2023). Changes of the subchondral bone microchannel network in early osteoarthritis. Osteoarthr. Cartil. 31 (1), 49–59. 10.1016/j.joca.2022.10.002 36243309

[B30] Temp J. Labuz D. Negrete R. Sunkara V. Machelska H. (2020). Pain and knee damage in Male and female mice in the medial meniscal transection-induced osteoarthritis. Osteoarthr. Cartil. 28 (4), 475–485. 10.1016/j.joca.2019.11.003 31830592

[B31] Tong L. Yu H. Huang X. Shen J. Xiao G. Chen L. (2022). Current understanding of osteoarthritis pathogenesis and relevant new approaches. Bone Res. 10 (1), 60. 10.1038/s41413-022-00226-9 36127328 PMC9489702

[B32] Wang W. Luan F. Shi Y. Zhang X. Guo D. Sun J. (2025). “Combination of UHPLC-QE-MS and network pharmacology to reveal the mechanism of Fufang-Duzhong-Jiangu granules for treating knee osteoarthritis,”, 39. Jan. 10.1002/bmc.6051 Biomed. Chromatogr. 1 e6051 39662515

[B33] Xian L. Wu X. Pang L. Lou M. Rosen C. J. Qiu T. (2012). Matrix IGF-1 maintains bone mass by activation of mTOR in mesenchymal stem cells. Nat. Med. 18 (7), 1095–1101. 10.1038/nm.2793 22729283 PMC3438316

[B34] Xie L. Tintani F. Wang X. Li F. Zhen G. Qiu T. (2016). Systemic neutralization of TGF-β attenuates osteoarthritis. Ann. N. Y. Acad. Sci. 1376 (1), 53–64. 10.1111/nyas.13000 26837060 PMC4970979

[B35] Xiong J. Onal M. Jilka R. L. Weinstein R. S. Manolagas S. C. O'Brien C. A. (2011). Matrix-embedded cells control osteoclast formation. Nat. Med. 17 (10), 1235–1241. 10.1038/nm.2448 21909103 PMC3192296

[B36] Yu D. Hu J. Sheng Z. Fu G. Wang Y. Chen Y. (2020). Dual roles of misshapen/NIK-related kinase (MINK1) in osteoarthritis subtypes through the activation of TGFβ signaling. Osteoarthr. Cartil. 28 (1), 112–121. 10.1016/j.joca.2019.09.009 31647983

[B37] Zhang P. Ye J. Dai J. Wang Y. Chen G. Hu J. (2022). Gallic acid inhibits osteoclastogenesis and prevents ovariectomy-induced bone loss. Front. Endocrinol. (Lausanne) 13, 963237. 10.3389/fendo.2022.963237 36601012 PMC9807166

[B38] Zhang W. Ouyang H. Dass C. R. Xu J. (2016). Current research on pharmacologic and regenerative therapies for osteoarthritis. Bone Res. 4, 15040. 10.1038/boneres.2015.40 26962464 PMC4772471

[B39] Zhao W. Wang T. Luo Q. Chen Y. Leung V. Y. Wen C. (2016). Cartilage degeneration and excessive subchondral bone formation in spontaneous osteoarthritis involves altered TGF-β signaling. J. Orthop. Res. 34 (5), 763–770. 10.1002/jor.23079 26496668

[B40] Zhen G. Fu Y. Zhang C. Ford N. C. Wu X. Wu Q. (2022). Mechanisms of bone pain: progress in research from bench to bedside. Bone Res. 10 (1), 44. 10.1038/s41413-022-00217-w 35668080 PMC9170780

[B41] Zhen G. Guo Q. Li Y. Wu C. Zhu S. Wang R. (2021). Mechanical stress determines the configuration of TGFβ activation in articular cartilage. Nat. Commun. 12 (1), 1706. 10.1038/s41467-021-21948-0 33731712 PMC7969741

[B42] Zhen G. Wen C. Jia X. Li Y. Crane J. L. Mears S. C. (2013). Inhibition of TGF-β signaling in mesenchymal stem cells of subchondral bone attenuates osteoarthritis. Nat. Med. 19 (6), 704–712. 10.1038/nm.3143 23685840 PMC3676689

[B43] Zhu J. Zhen G. An S. Wang X. Wan M. Li Y. (2020). Aberrant subchondral osteoblastic metabolism modifies Na(V)1.8 for osteoarthritis. Elife 9, e57656. 10.7554/eLife.57656 32441256 PMC7308086

[B44] Zhu S. Zhu J. Zhen G. Hu Y. An S. Li Y. (2019). Subchondral bone osteoclasts induce sensory innervation and osteoarthritis pain. J. Clin. Investig. 129 (3), 1076–1093. 10.1172/JCI121561 30530994 PMC6391093

