# Supplementary material for: Fufang Duzhong Jiangu granule (FFDZ) ameliorates osteoarthritis development through maintaining subchondral bone homeostasis
Source: Front Cell Dev Biol. 2025 Aug 6;13:1610007. doi: 10.3389/fcell.2025.1610007 (PMC12364815; doi:10.3389/fcell.2025.1610007)
Supplement: Supplementary file 1 [file DataSheet1.doc]

**Supplementary Data**

**Supplementary Table 1 The** **main components of FFDZ.**

| **Drug name** | **Latin name** |
| --- | --- |
| Duzhong | *Eucommia ulmoides* Oliv. |
| Baishao | *Paeonia lactiflora* Pall. |
| Xuduan | *Dipsacus asper* Wall. |
| Gouqizi | *Lycium barbarum* L. |
| Niuxi | *Achyranthes bidentata* Bl. |
| Sanqi | *Panax notoginseng* Burk. |
| Jixueteng | *Spatholobus suberectus* Dunn. |
| Renshen | *Panax ginseng* C. |
| Danggui | *Angelica sinensis* Oliv. |
| Huangbai | *Phellodendron chinense* Schneid. |
| Weilingxian | *Clematis chinensis* Osbeck. |


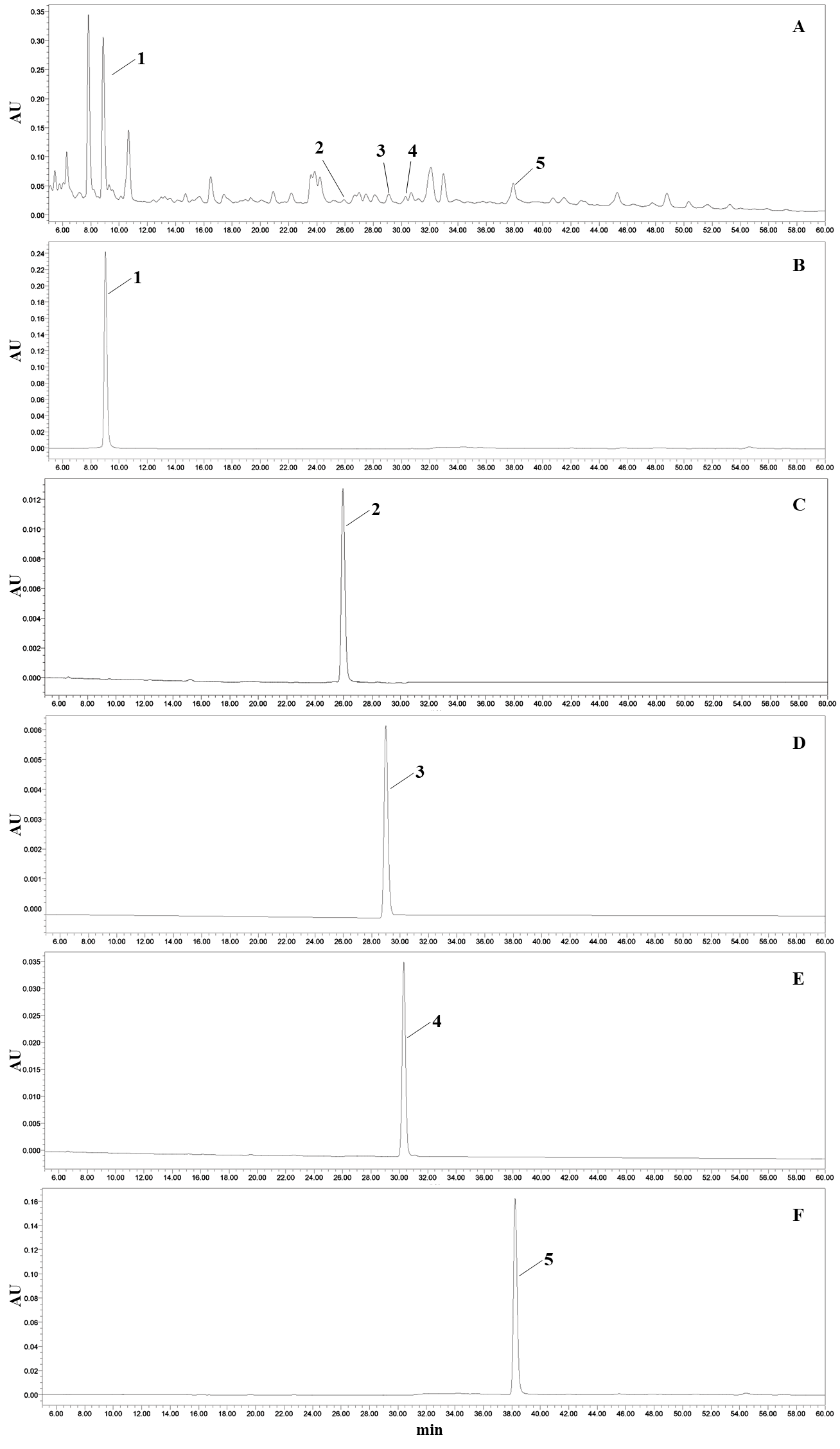


Supplementary Fig. 1. Chromatographic fingerprint analysis of FFDZ by UPLC. (A) Chromatogram of FFDZ extract. (B-F) Chromatogram of five standard chemicals: (1) gallic acid, (2) geniposide, (3) geniposide, (4) pinecrosinol diglucoside, (5) calycosin-7-glucoside).


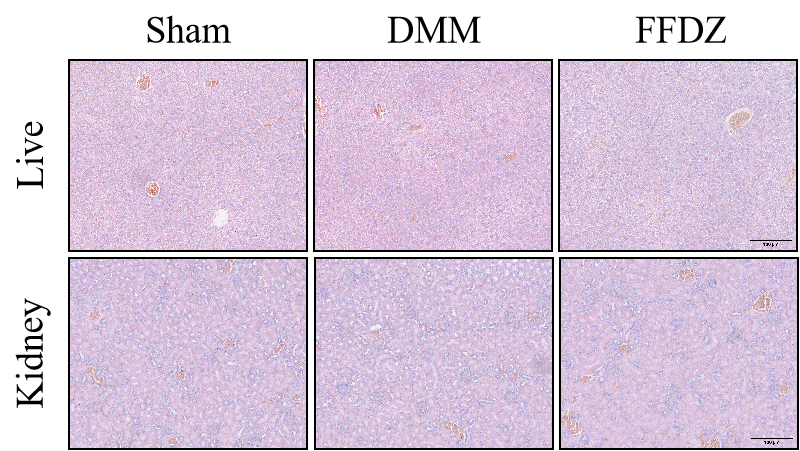


Supplementary Fig. 2 H&E staining of mouse liver and kidney tissues


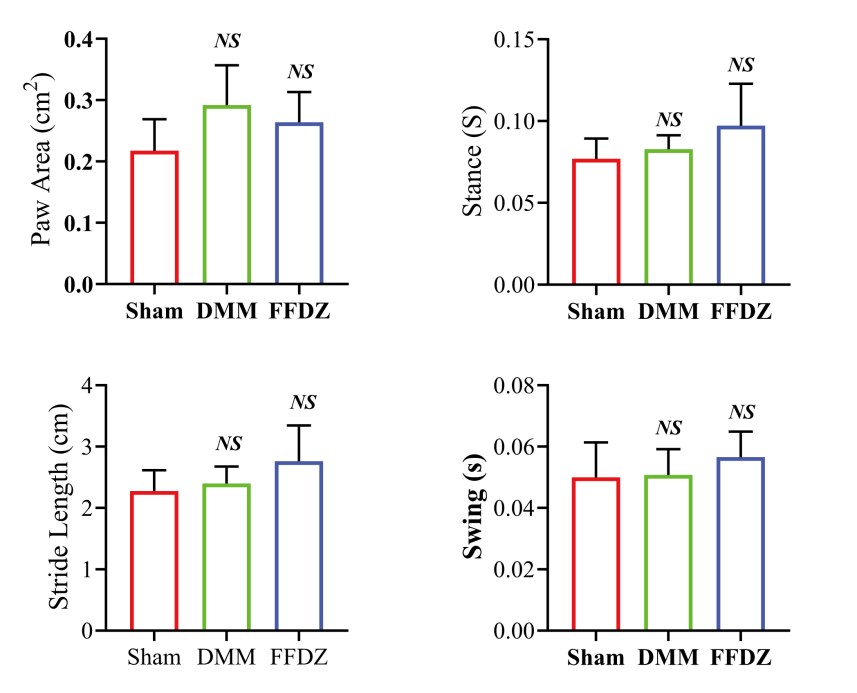


Supplementary Fig. 3 Gait Analysis in 4-week Mice


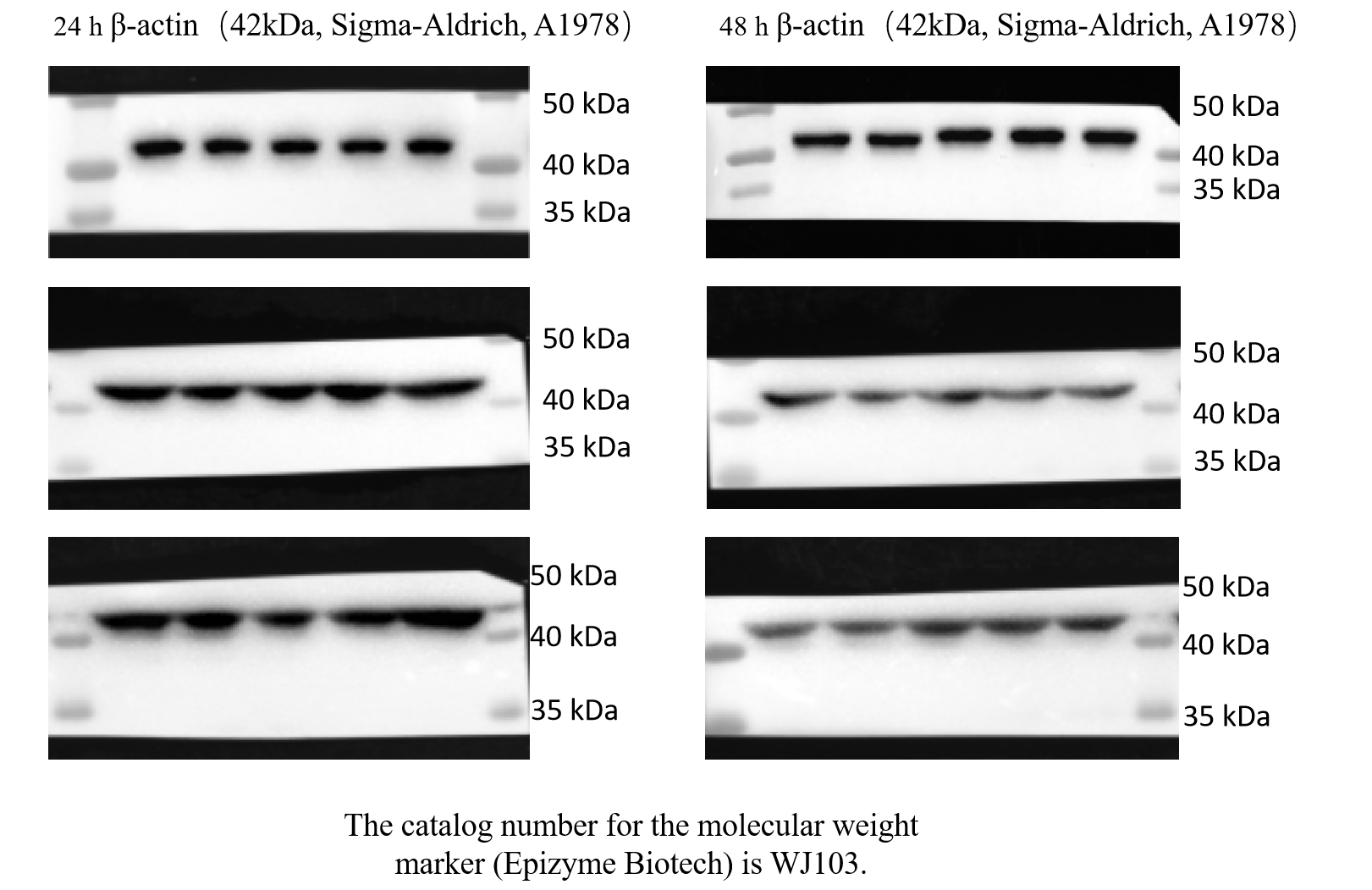

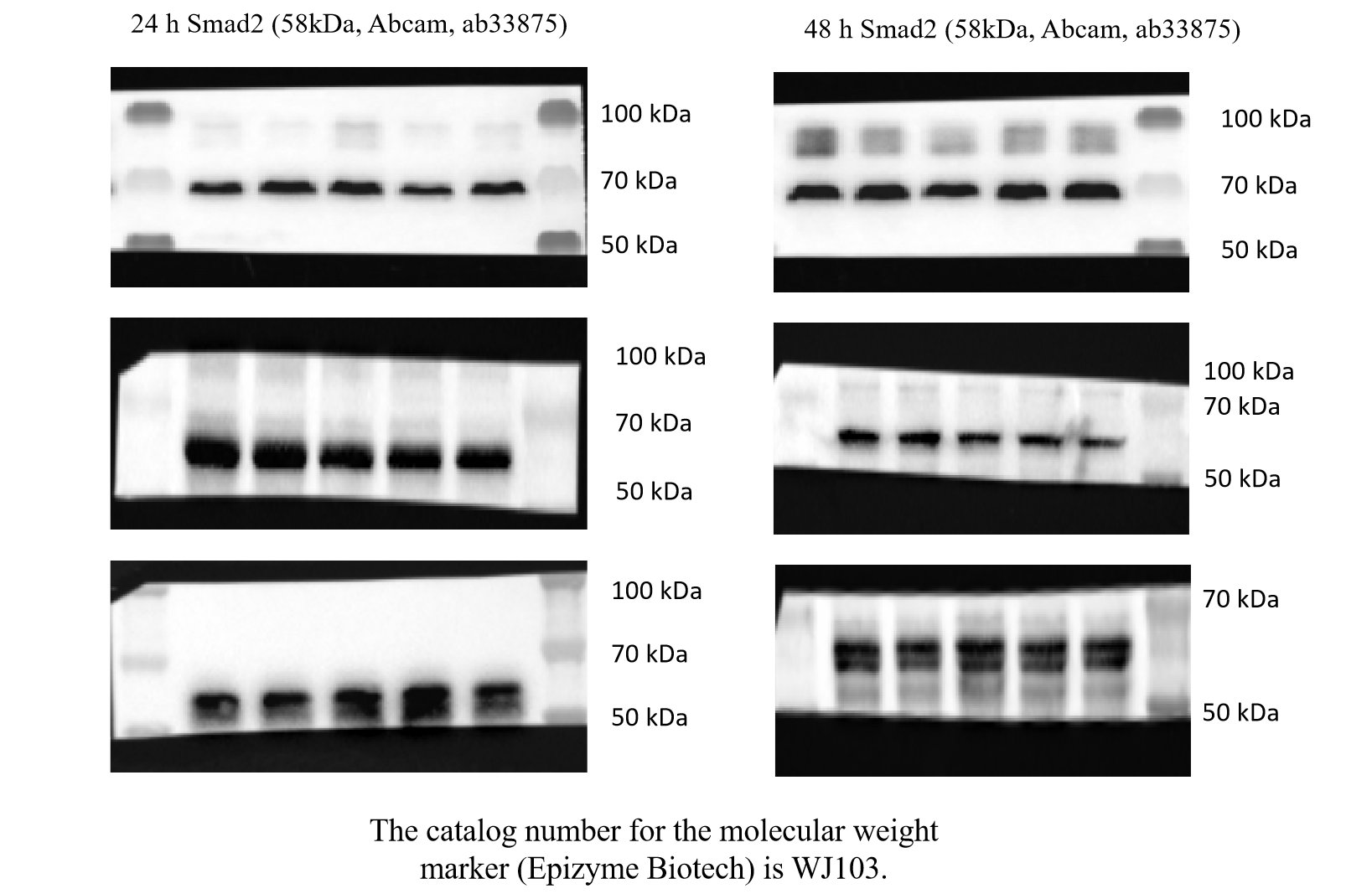

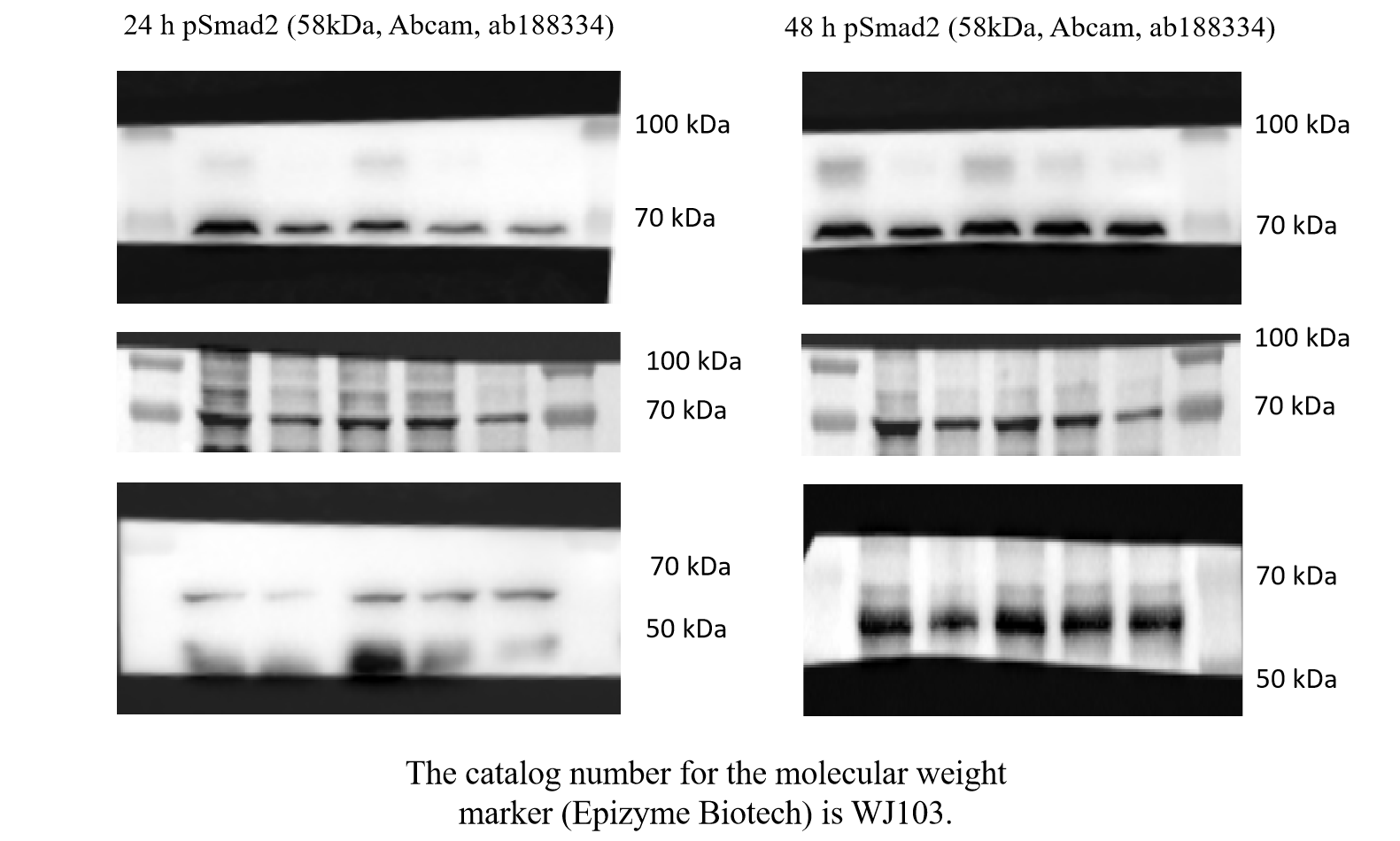


Supplementary Fig. 4 Western blot replicate experiments
